# Adjunctive GM-CSF therapy enhances host defense against systemic *Candida auris* infection in immunosuppressed mice

**DOI:** 10.3389/fimmu.2025.1731315

**Published:** 2026-01-19

**Authors:** Eliciane Cevolani Mattos, Kaustav Das Gupta, Derek Quintanilla, Haley Hautau, Ashraf S. Ibrahim, Shakti Singh

**Affiliations:** 1 Division of Infectious Disease, The Lundquist Institute for Biomedical Innovation at Harbor–University of California, Los Angeles Medical Center, Torrance, CA, United States; 2 David Geffen School of Medicine, University of California, Los Angeles, Los Angeles, CA, United States

**Keywords:** Immunosuppression, murine systemic infection model, antifungal immunity, *Candida auris*, GM-CSF, immunotherapy, macrophage, neutrophils

## Abstract

**Introduction:**

*Candida auris* is an emerging, multidrug-resistant fungal pathogen associated with high mortality in immunocompromised individuals. Resistance to current antifungal drugs emphasizes the need for new therapeutic approaches. We investigated granulocyte-macrophage colony-stimulating factor (GM-CSF) as a standalone immunotherapy and in combination with a sub-therapeutic dose of micafungin in an immunosuppressed mouse model of systemic *C. auris* infection.

**Methods:**

Immunosuppressed ICR CD-1 mice (4–6 weeks old) were infected with *C. auris* and treated daily via intraperitoneal injection with PBS (placebo), murine GM-CSF (0.2 or 2 μg), micafungin, or both. Treatments began 24 h post-infection and continued through day 4 (GM-CSF) or day 7 (micafungin). Survival, tissue fungal burden, histopathology, and immune cell frequencies in spleen and kidneys were assessed. GM-CSF effects on neutrophil and macrophage antifungal functions were evaluated in *ex vivo* assays.

**Results:**

GM-CSF monotherapy significantly improved survival (30–32% vs. 0% in controls), extended median survival time, and reduced fungal burden in the kidney, heart, and brain. Although the combination therapy yielded the highest survival rate, it did not differ significantly from GM-CSF alone. Histopathological examination confirmed decreased fungal load and tissue damage in GM-CSF-treated mice. Additionally, GM-CSF augmented macrophage and neutrophil populations in spleen and kidney, enhanced fungal uptake and killing via reactive oxygen species and neutrophil extracellular traps.

**Discussion:**

GM-CSF augments antifungal immunity and represents a promising adjunctive immunotherapy against MDR *C. auris* infection.

## Introduction

*Candida auris* is an emerging fungal pathogen that poses a significant threat to public health due to its multidrug resistance (MDR) and high mortality rates (~60%), particularly among immunocompromised individuals (1). First identified in 2009 in Japan, *C. auris* has since been reported in over 40 countries, often causing outbreaks in healthcare settings (2, 3). *C. auris* can colonize human skin and cause severe, life-threatening bloodstream infections (2). Additionally, *C. auris* can persist on surfaces in healthcare environments, facilitating its spread among patients and posing a risk to immunocompromised patients harboring invasive medical devices (2, 4).

One of the significant challenges in managing *C. auris* infections is its resistance to multiple antifungal classes, including azoles, echinocandins, and polyenes (1, 2, 5). Investigations of available *C. auris* clinical isolates showed that ~90% are resistant to azoles, 35% to polyenes, and 5% to echinocandins (1, 6, 7). The resistance to clinically available antifungals complicates treatment and increases the risk of treatment failure. Several novel antifungals, such as ibrexafungerp and fosmanogepix, have shown promising antifungal activity against *C. auris* in early pre-clinical studies (8, 9), and their efficacy is now being tested in clinical trials (9, 10). Despite these new developments, the emergence of resistant *C. auris* isolates will likely continue. Thus, unconventional adjunctive immunotherapies are a promising alternative approach to addressing multidrug-resistant pathogens.

Granulocyte-macrophage colony-stimulating factor (GM-CSF) is a pleiotropic cytokine that plays a crucial role in antifungal immune defense by promoting the differentiation of phagocytes, including neutrophils, macrophages, and dendritic cells. *C. auris* is known to induce a weaker immune response *in vivo* compared to *C. albicans* (11). Thus, the immunomodulatory effect of GM-CSF can be beneficial for activating antifungal immune responses (12–14), particularly in patients with weakened immune systems, such as those undergoing chemotherapy or organ transplantation. GM-CSF, as a combination therapy, can also help reduce the dosage and duration of antifungal medications, potentially minimizing their side effects and maximizing their protective efficacy (15, 16).

GM-CSF biologics, such as sargramostim, have been approved by the US FDA for indications including neutropenia following bone marrow transplantation and chemotherapy, and for progenitor cell mobilization for stem cell collection (17). A few studies have explored GM-CSF’s potential as an adjunctive therapy in treating fungal infections, particularly in immunocompromised patients. GM-CSF, when used as an adjunct therapy to antifungal drugs, has shown synergistic effects and improved patient outcomes by enhancing the immune response and reducing fungal burden in immunocompromised patients with disseminated *Scedosporium, Histoplasma, Cryptococcus*, and invasive aspergillosis (15, 18–22). Further, reports of GM-CSF therapy for oropharyngeal candidiasis and endocarditis caused by *C. parapsilosis* have only limited benefits (23, 24).

Despite the MDR nature of *C. auris*, the protective benefits of GM-CSF monotherapy and combination therapy with first-line therapy, echinocandins, haven’t been explored. Thus, we sought to test the potential of GM-CSF as an immunotherapeutic agent against MDR *C. auris* infection. Using a clinically relevant immunosuppressed murine infection model, we investigated the efficacy of GM-CSF in improving overall survival, reducing fungal burden, and mitigating tissue damage in infected mice. Our research aimed to provide insights into the viability of GM-CSF as an adjunctive therapy. We also investigated the immunomodulatory effects of GM-CSF on innate antifungal immune responses, which could enhance the effectiveness of existing antifungal treatments and offer a new multi-pronged strategy to combat MDR *C. auris* infections.

## Materials and methods

### Isolates, growth conditions, and inoculum preparation

In this study, we utilized the *C. auris* CAU-09 (South Asian, clade I) and *C. albicans* SC5314 strain (25). For inoculum preparation, both the yeasts were cultured overnight in Yeast Extract Peptone Dextrose (YPD) broth at 30°C with continuous shaking at 200 rpm. Following incubation, the yeast cells were washed three times with 1x phosphate-buffered saline (PBS, Cat: 10010023, Thermo Fisher Scientific). The blastoconidia were then counted using a hemocytometer and adjusted to the desired cell density (25).

### Mice infection and treatment

We used outbred ICR CD-1 male mice in this study, aged 4 to 6 weeks and weighing 25–30 grams. The mice were purchased from Envigo Harlan in Seattle, WA, USA. To induce immunosuppression, the mice received intraperitoneal (I.P.) injections of 200 mg cyclophosphamide and subcutaneous (S.C.) injections of 250 mg cortisone 21-acetate (Cat: C3130, Millipore Sigma) per kilogram (kg) of body weight, administered two days before infection. To prevent bacterial superinfection, the antibiotic, enrofloxacin (Baytril^®^, Elanco, Germany) was added to the drinking water at 50 µg/ml, starting the same day and continuing until day 10 post-infection (25–27).

The immunosuppressed mice were then intravenously (I.V.) infected with a lethal dose of 5 × 10^7^ (for survival studies) or a sublethal dose of 1 × 10^7^ (for fungal burden studies) of *C. auris* (CAU-09) cells/mouse. For *C. albicans*, immunosuppressed mice were infected with a lethal I.V. dose of 5 × 10^4^ yeast cells/mouse. The inoculum size for each *Candida* species was determined based on previously published research and preliminary experiments to achieve comparable disease severity (25, 27). Because *C. albicans* is lethal at an inoculum ~2 logs lower than that of *C. auris*, even in immunocompetent mice, its inoculum was adjusted to produce a survival curve similar to that of untreated mice infected with *C. auris*, ensuring clinical relevance and consistency across models (25, 28).

One day post-infection, the mice were randomly assigned to the following non-blinded treatment groups: 0 (PBS diluent or placebo, n=10 + 9 mice, from two experiments), 0.2 (n=10 mice), 2.0 µg/mouse GM-CSF (Cat: Z03300, GenScript) (n=10 + 9 mice, from two experiments) or 0.5 mg/kg/day of micafungin (n=10 mice) or 2.0 µg/mouse GM-CSF + 0.5 mg/kg/day of micafungin (n=9 mice). Uninfected immunosuppressed mice served as a control for any unintended mortality caused by opportunistic bacterial infections. GM-CSF was administered I.P. once daily from day 1 through day 4. A subtherapeutic daily dose of micafungin was initiated on day 1 post-infection and continued until day 7. The mice were monitored for survival over 21 days post-infection (25, 27, 29).

For fungal burden, mice were euthanized on day 4 post-infection to collect kidneys, hearts, and brains. The organs were weighed, homogenized, and quantitatively cultured using 10-fold serial dilutions on YPD plates. The plates were incubated at 37 °C for 48 hours before colony-forming units (CFUs) per gram of tissue were enumerated (25, 27).

For the histopathological examination, kidneys and hearts from representative mice were harvested on days 4 and 7 post-infection. The organs were fixed in 10% zinc-buffered formalin, embedded in paraffin, sectioned, and stained with Hematoxylin and Eosin (25–27, 29).

### Isolation of bone marrow neutrophils and peritoneal macrophages

Immunocompetent ICR CD-1 mice were injected with 2.0 µg/mouse I.P. of GM-CSF or PBS once daily for up to 4 days. Post euthanization on day 4, peritoneal exudates and bone marrow cells were harvested from each mouse. Bone marrow neutrophils were purified using the MojoSort Mouse Neutrophil Isolation Kit (Cat: 480058, BioLegend) according to the manufacturer’s instructions. The purity of neutrophils was assessed using flow cytometry as described below. Bone marrow neutrophils and cells from peritoneal exudates were then evaluated using the assays described below.

### *In vitro* infection assay

Approximately 1x10^6^ bone marrow neutrophils and peritoneal exudates were plated on 24-well plates in complete RPMI-1640 media (Cat: R8758, Millipore Sigma) supplemented with 10% FBS (Cat: A5670801, Thermo Fisher Scientific) and 1% Pen-Strep antibiotic cocktail (Cat: 15140122, Thermo Fisher Scientific). The cells were allowed to settle and adhere for two hours before infecting them with CAU-09 (MOI 1) for four hours. To determine the percentage killing, CAU-09 was grown for four hours in complete RPMI media without host cells. Host neutrophils and peritoneal exudate cells were lysed using 0.05% Triton X-100. We enumerated fungal cells after incubation with and without host cells by plating the fungus on YPD agar plates and counting CFU the following day (30). The percentage killing of *C. auris* was calculated and compared.

Approximately 2.5 x 10^5^ RAW264.7 macrophages (ATCC) were plated on a 24-well plate in complete RPMI media and treated with 20 ng/ml GM-CSF overnight. The following day, the macrophages were infected with CAU-09 (MOI 1) for 4 hours. Percentage killing was determined and compared as described above.

### Determination of neutrophil elastase activity

Approximately 1 × 10^6^ bone marrow neutrophils were infected with CAU-09 (MOI 1) for four hours. Post-infection, the neutrophils were centrifuged at 500 g for 5 minutes, and the supernatant was collected. Neutrophil elastase activity was determined using a mouse neutrophil elastase ELISA kit (Cat: ab252356, Abcam) according to the manufacturer’s instructions.

### Determination of cell cytotoxicity

Cytotoxicity of RAW264.7 cells, bone marrow neutrophils, and cells from peritoneal exudates upon infection with CAU-09 (MOI 1, 4 hours) was assessed by measuring lactate dehydrogenase in the cell culture supernatant using the CytoTox 96 Non-Radioactive Cytotoxicity Assay kit (Cat: G1780, Promega), following the manufacturer’s instructions.

### Phenotyping of immune cells by flow cytometry

Mouse spleens and kidneys from infected and GM-CSF-treated mice were collected on days 4 and 7 and processed individually by homogenization through a 100 μm cell strainer. Red blood cells (RBCs) were lysed using 1x RBC lysis buffer (Cat: SC-296258, Santa Cruz Biotech, Dallas) and filtered through 100 μm sterile filters (25). Splenocytes and Kidney cells were stained with antibodies against CD3, CD4, CD19, F4/80, CD11b, and Ly6G. To determine the purity of neutrophils and peritoneal macrophages, the cells were stained using Ly6G, CD11b, and F4/80. The details of the antibodies used are provided in **Supplementary Table S4**. The stained cells were acquired using a BD LSR II flow cytometer, and the data were analyzed with FlowJo V10.

### Flow cytometry-based determination of fungal uptake

An overnight culture of CAU-09 was washed twice with PBS, then manually counted on a hemocytometer to adjust the MOI to 1. Fungal cells were stained with 10 μM CellTrace CFSE Cell Proliferation Kit (Cat: C34554, Thermo Fisher Scientific) for 45 minutes at 37 °C. Post-staining, the fungal cells were washed twice with PBS to remove any additional dye before infecting host cells (RAW264.7 macrophages (pre-treated overnight with 20 ng/ml GM-CSF), bone marrow neutrophils, and cells from peritoneal exudates) for 1.5 hours. The host cells are washed twice with PBS to remove any planktonic fungus, then lifted using a lift buffer (PBS containing 2% FBS, 0.5 mM EDTA, and 0.1% sodium azide). Fungal uptake by host cells was then assessed using flow cytometry (31).

### Determination of reactive oxygen species using flow cytometry

An overnight culture of CAU-09 was washed twice with PBS and then manually counted to adjust the MOI to 1. RAW264.7 macrophages were pre-treated with 20 ng/ml GM-CSF overnight. Host cells (RAW264.7 macrophages, bone marrow neutrophils, and cells from peritoneal exudates) were pre-treated with 10 μM CM-H_2_DCFDA (Cat: C6827, Thermo Fisher Scientific) for 30 minutes at 37 °C. Post-staining, the host cells were washed twice with PBS to remove excess dye, then infected with CAU-09 for 1.5 hours. The cells are washed twice with PBS to remove any planktonic fungus, then lifted using the Lift buffer. ROS generation upon fungal infection was then assessed using flow cytometry (32).

### Cytokine analysis

For cytokine analysis, the spleens were collected from euthanized mice on day 4 post-infection and homogenized in PBS containing a Protease Inhibitor cocktail (Cat: 78425, Thermo Fisher Scientific). Cytokine concentrations in splenic lysates were assessed using the Mouse Magnetic Luminex Assay Kit (Cat: LXSAMSM, R&D Systems) according to the manufacturer’s instructions, and analyzed using the Luminex system (Invitrogen).

### Statistical analysis

The mouse groups were compared by the log-rank (Mantel-Cox) test for survival studies. In these studies, at least nine mice per group were used based on prior experience with the *C. auris* infection model, which results in 100% mortality when untreated, and on a power analysis for a two-arm survival comparison (placebo vs. treatment) using the log-rank (Mantel-Cox) test. This sample size provided 90% power by assuming a 50% survival benefit in the treatment group (Hazard Ratio = 0.5), one-sided α = 0.05, equal allocation, and complete event occurrence. Selected treatment groups were repeated, and data from replicates were pooled for analysis. No confounders were considered in the study, since all immunosuppressed uninfected mice appeared healthy.

Sample size calculations for fungal burden experiments assumed α = 0.05, 90% power, and an effect size of at least 1.2 log CFU, resulting in an estimated sample size of approximately 10 mice per group. We used 11 mice per group to ensure we have sufficient surviving mice at the time of fungal enumeration on day 4 post-infection.

Percent weight loss of mice (**Supplementary Figure S1B**), tissue fungal burden, immune cell functional activity, and cytokine analysis (**Supplementary Figure S6**) data are graphically presented as median ± interquartile range (IQR). Statistical significance between the two treatment groups was assessed using the Mann-Whitney test. For multiple comparisons among groups, we used the Kruskal-Wallis test followed by Dunnett’s correction for cytokine analysis, and a 2-way ANOVA (Tukey’s test) for elastase activity. These statistical tests were used to nullify the effects of outliers, making them suitable for small sample sizes.

Power analysis was not conducted for experiments involving the determination of immune cell frequency. The data for immune cell phenotyping (**Supplementary Figures S2, S4, S5**) were presented as bar graphs with each data point representing an independent biological replicate (3 per group), ensuring independence between groups. Because comparisons involved two independent groups without a directional hypothesis, we used an unpaired, two−tailed Student’s t−test to assess differences in means. Data are shown as mean ± SEM to indicate precision, and the t−test is appropriate under the assumptions of approximate normality and equal variances, which are reasonable for these aggregated phenotyping measures.

Graphical representations and statistical analyses of numerical data were performed using GraphPad Prism (v.10.4). Detailed descriptive statistics are presented as part of the main figure or separately (**Supplementary Table S1-3**) under the **supplementary file**. Histopathological images are representative of >3 biological replicates.

### Ethics statement

Mice were housed in the Lundquist Institute (TLI) pathogen-free animal facility until infection, and all animal procedures were approved by and in compliance with the TLI IACUC guidelines (Protocol # 2024-33082-01).

### Compliance with ARRIVE guidelines

The study design, data presentation, statistical analysis, and reporting were conducted in accordance with the ARRIVE (Animal Research: Reporting of *In Vivo* Experiments) guidelines to ensure transparency, reproducibility, and ethical standards in animal research (33).

## Results

### GM-CSF monotherapy and antifungal combination therapy protect mice against lethal hematogenously disseminated *C. auris* but not *C. albicans* infection

**Figure 1:**
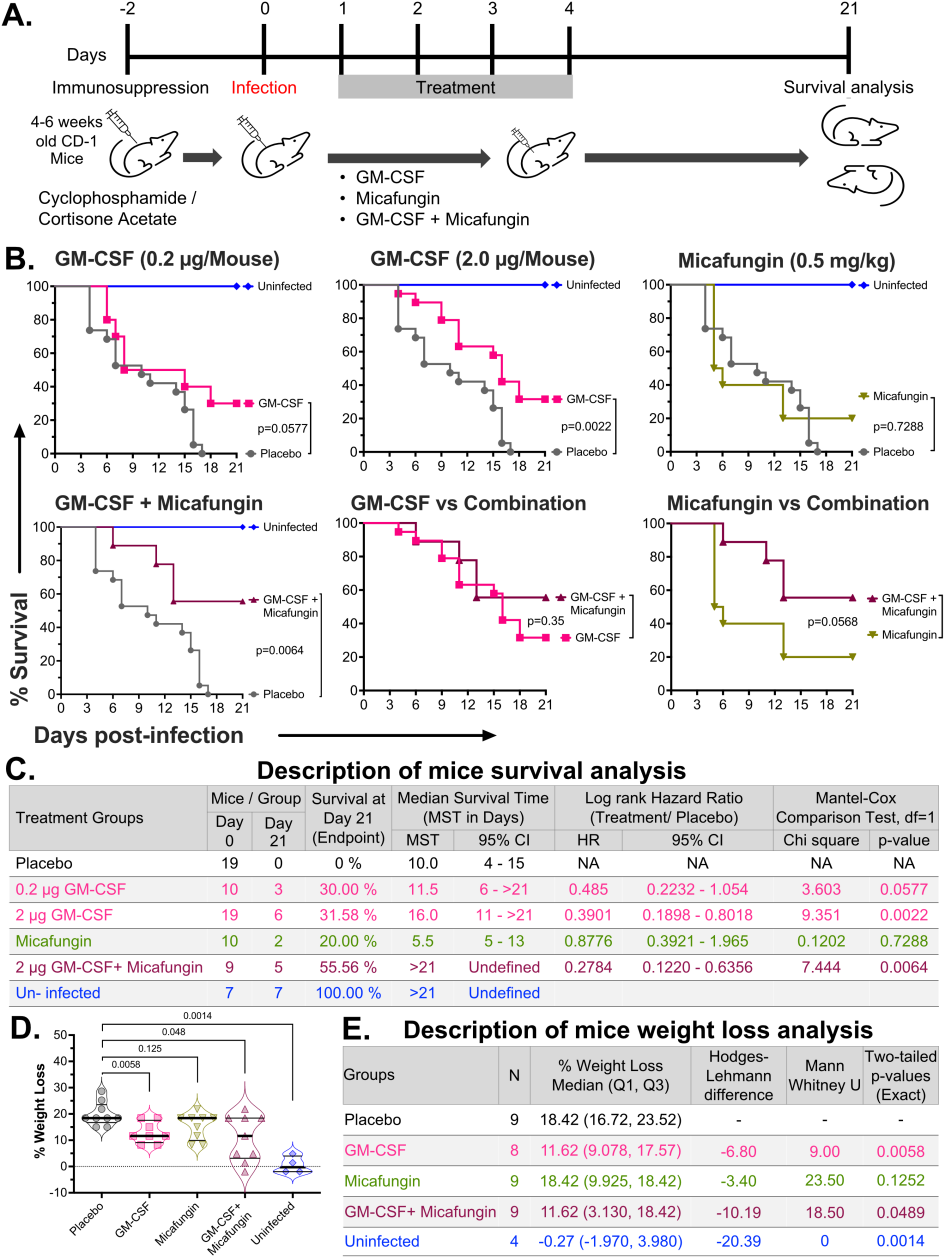
Efficacy of GM-CSF monotherapy and antifungal combination therapy against lethal *C. auris* systemic infection in immunosuppressed mice. **(A)** 4–6 weeks old immunosuppressed ICR CD-1 mice were infected with a lethal inoculum of *C. auris* (5 x 10^7^ cells/mouse), and treated with PBS diluent (placebo), 0.2 μg murine GM-CSF, 2 μg murine GM-CSF, micafungin, or both via daily intraperitoneal injections. The GM-CSF and micafungin treatments were initiated 24 hours post-infection and continued through days 4 and 7, respectively. **(B)** Mice survival was compared after 21 days post-infection by the Mantel-Cox test. **(C)** The table describes the animal number/treatment group, percent survival, median survival time (with 95% confidence intervals [CI]), hazard ratios versus the placebo group (with 95% CI), and p-values for the Mantel-Cox test for treatment vs placebo. **(D)** Mice percent weight loss was compared between treatment groups at day 4 post-infection using the Mann-Whitney test, and results were expressed as median ± interquartile range (IQR), and a two-tailed p<0.05 was considered significant. **(E)** Descriptive statistics of mice weight loss analysis.

Since *C. auris* causes lethal bloodstream infections in immunosuppressed patients, we utilized a clinically relevant cyclophosphamide and cortisone acetate-induced immunosuppressed mouse model in an outbred ICR CD-1 background. We hematogenously infected these mice via tail vein injection with a lethal inoculum (5 x 10^7^ yeast/mouse) of *C. auris* (CAU-09) or (5 x 10^4^ cells/mouse) of *C. albicans* (SC5314) after 2 days of immunosuppression. Mice were treated with intraperitoneal injection of GM-CSF once daily for up to four days, starting 24 hours post-infection. All infected mice were randomly divided into different treatment groups. Treatment efficacy and mouse health were evaluated by monitoring survival for 21 days and body weight loss at day 4 post-infection. No mice were excluded from the analysis (**Figure 1A**, **Supplementary Figure S1A**).

Twenty-one-day survivals of mice were analyzed for each treatment group using Kaplan-Meier analysis. All placebo (diluent vehicle)-treated mice (n=19, pooled from two independent experiments) succumbed to infection before day 21, with 0% survival and a median survival time (MST) of 10 days (95% CI: 4–15). A low dose of GM-CSF (0.2 µg/mouse, n=10) improved survival to 30% with a median survival of 11.5 days (95% CI: 6 – >21), showing a trend toward significance compared to placebo (HR = 0.485, 95% CI: 0.2232–1.054; p = 0.0577). Consistent with this trend, a high-dose of GM-CSF (2.0 µg/mouse, n=19 pooled from two independent experiments) further enhanced survival to 32% and extended median survival to 16 days (95% CI: 11 – >21), achieving statistical significance versus placebo (HR = 0.3901, 95% CI: 0.1898–0.8018; p = 0.0022) (**Figures 1B, C**).

We also evaluated whether the GM-CSF therapy synergizes with an approved antifungal drug. A sub-therapeutic dose (0.5 mg/kg body weight) of micafungin combined with GM-CSF (2 μg GM-CSF/mouse, n=9) was tested. Micafungin monotherapy (n=10) demonstrated limited benefit, with 21-day survival of 20% and an MST of 5.5 days (95% CI: 5–13), which was not significantly different from placebo (HR = 0.8776, 95% CI: 0.3921–1.965; p = 0.7288). Combination therapy with GM-CSF and micafungin demonstrated the most significant improvement, with 55.6% survival at day 21 and a median survival of >21 days. This regimen significantly reduced mortality risk compared to placebo (HR = 0.2784, 95% CI: 0.122–0.6356; p = 0.0064). The survival benefit of combination therapy did not reach statistical significance compared with GM-CSF (χ² = 0.8857, p = 0.3466) or micafungin monotherapy (χ² = 3.629, p = 0.0568) (**Figures 1B, C**).

*C. auris*-infected mice lose weight as the infection progresses, which reflects the overall health of the mice. Thus, we compared weight loss among different treatment groups after 4 days of infection. The placebo-treated mice (n=9) showed the highest weight loss (Median:18.42%, IQR: 16.72 – 23.52), followed by micafungin (n=9, Median: 18.42%, IQR: 9.925 – 18.42), GM-CSF (n= 8, one mouse died before day 4 weight measurement, Median: 11.62%, IQR: 9.078 – 17.57), and combination therapy (n=9, Median: 11.62%, IQR: 3.130 –18.42). Thus, compared to placebo, both GM-CSF monotherapy (U = 9, two-tailed exact p=0.0058) and antifungal combination therapy (U = 18.50, two-tailed exact p=0.0489) showed significantly less weight loss due to *C. auris* infection, demonstrating superior protective efficacy compared to the placebo-treated group (**Figures 1D, E**).

Interestingly, GM-CSF therapy failed to prevent mortality in *C. albicans-*infected mice (n=9/group), but lost significantly less weight (n=3, median: 11.11%, IQR: 5.56 – 13.89) vs placebo (n=5, median: 19.77%, IQR: 16.91– 21.2) in surviving mice at day 3 post infection (U = 0, two-tailed exact p=0.0179) (**Supplementary Figures S1B, C**).

### GM-CSF therapy controlled the tissue *C. auris* burden and host organ tissue damage

**Figure 2:**
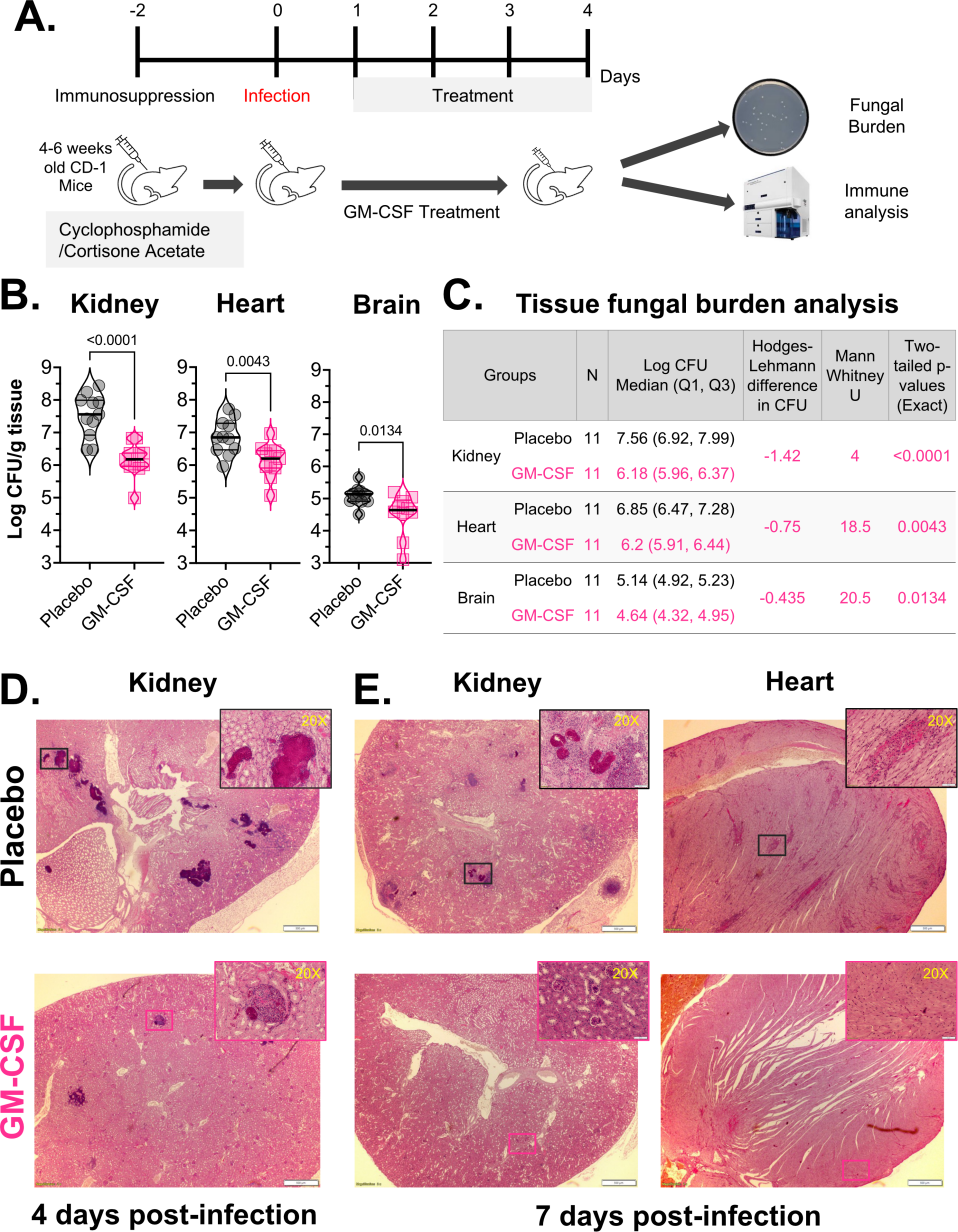
Tissue *C. auris* burden and histopathology analysis of infected immunosuppressed mice after GM-CSF immunotherapy. **(A)** 4–6 weeks old immunosuppressed ICR CD-1 mice (n =11/group) were infected with a sub-lethal inoculum of *C. auris* (1 x 10^7^ cells/mouse). Mice were treated with murine GM-CSF (2.0 μg/mouse) through daily intraperitoneal injections, starting 24 hours post-infection and continuing up to 96 hours. **(B)** Fungal burden was enumerated in the kidney, heart, and brain on day 4 post-infection (4 hours after final treatment) and expressed as the median ± IQR. Statistical comparisons were made using the Mann-Whitney test, and p<0.05 was considered significant. **(C)** Descriptive statistics of tissue fungal burden analysis. **(D)** Histopathology of the kidney after 4 days of infection. **(E)** Histopathology of the kidney and heart after 7 days of infection. Kidney and heart sections from the representative mice were stained with H&E and imaged using an Olympus Microscope. White boxes represent infection foci (20X zoom in-set boxes).

We further investigated fungal control in target organ tissues by evaluating fungal burden after 4 days of GM-CSF therapy. Immunosuppressed ICR CD-1 mice infected with a sub-lethal 1 × 10^7^ yeast/mouse inoculum of *C. auris* were randomly assigned to two treatment groups: placebo or GM-CSF therapy. In this model, mice were treated with 2 μg/mouse of GM-CSF once daily, starting 24 hours post-infection and continuing for up to 96 hours, after which the target organs of kidneys, heart, and brain were harvested and processed for fungal enumeration and histopathological examination (**Figure 2A**).

The GM-CSF treatment significantly reduced median *C. auris* fungal burden in the mice’s kidneys by 1.42 log (Hodges-Lehmann difference, U = 4, two-tailed exact p<0.0001), hearts by 0.75 log (U = 18.5, two-tailed exact p=0.0043), and brains by 0.435 log (U = 20.5, two-tailed exact p=0.0134) per gram tissue when compared to placebo, respectively (**Figures 2B, C**). The histopathological examination corroborated these data on tissue fungal burden. Specifically, mice treated with GM-CSF had significantly fewer and smaller fungal abscesses in the kidney at day 4 and in both the kidney and the heart at day 7 post-infection. Furthermore, compared to day 4, the fungal lesions in kidney tissues were reduced even further by day 7. Additionally, GM-CSF-treated mice showed less necrosis and tissue damage than the placebo-treated mice, as indicated by visually more intact kidney and heart tissue architecture (**Figures 2D, E**).

### GM-CSF treatment modulates immune cell population and their antifungal responses in uninfected and *C. auris*-infected mice

**Figure 3:**
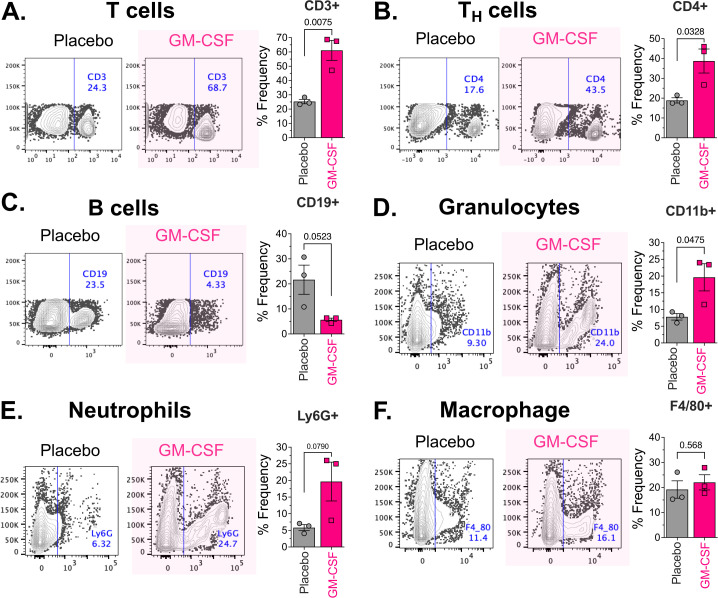
Effect of GM-CSF treatment on the spleen immune cell population in uninfected mice. Immunosuppressed ICR CD-1 mice (n=3/group) were treated with a 2 μg/mouse dose of murine GM-CSF once daily. After 4 days of treatment, the spleen of the mice was processed to prepare a single-cell suspension and stained with fluorescently labeled antibodies for specific immune cell lineage markers: **(A)**. CD3 for T cells, **(B)**. CD4 for T helper cells, **(C)**. CD19 for B cells, **(D)**. CD11b for Granulocytes, **(E)**. Ly6G for Neutrophils, and **(F)**. F4/80 for Macrophages. Isotype-matched antibodies were used as controls for gating the negative and positive populations. The stained cells were analyzed on a BD FACSymphony, and the data were analyzed in FlowJo v10. Representative scatter graphs and corresponding bar graphs (mean ± SEM) **(A-F)** are shown for the placebo and GM-CSF treatment groups. Significant differences in the immune cell populations were analyzed by Student’s t-tests (two-tailed), and p-values <0.05 were considered statistically significant.

We also studied the effect of GM-CSF (2 μg/mouse) on the spleen immune cell population in uninfected and infected mice. Treatment of uninfected mice with GM-CSF for 4 days significantly increased the populations of CD3^+^ T cells (mean difference: 35.80, 95% CI: 15.88 – 55.72, two-tailed t-test p=0.0075) and CD4^+^ T helper cells (mean difference: 19.87, 95% CI: 2.649 – 37.08, two-tailed t-test p=0.0328) vs placebo treated mice (**Figures 3A, B**; **Supplementary Figures S2A, B**, two-tailed p<0.05). We also found an increase in CD11b^+^ granulocytes (mean difference: 11.82, 95% CI: 0.207 – 23.43, two-tailed t-test p=0.0475) in GM-CSF-treated mice (**Figure 3D**, **Supplementary Figure S2D**), which was generally accompanied by a strong trend toward increased neutrophils (mean difference: 13.85, 95% CI: -2.555 – 30.26, two-tailed t-test p=0.079) vs placebo-treated mice (**Figure 3E**, **Supplementary Figure S2E**). The CD19^+^ B cells (mean difference: -16, 95% CI: -32.26 – 0.2577, two-tailed t-test p=0.0523) and macrophage population (mean difference: 2.867, 95% CI: -9.956 – 15.69, two-tailed t-test p=0.5684) in the spleen were not changed significantly by GM-CSF treatment (**Figures 3C, F**; **Supplementary Figures S2C, F**). Interestingly, four days of GM-CSF treatment also increased the neutrophil population in the bone marrow (**Supplementary Figure S3A**) and both the neutrophil and macrophage populations in the intraperitoneal cavity in immunocompetent mice (**Supplementary Figure S3B**).

**Figure 4:**
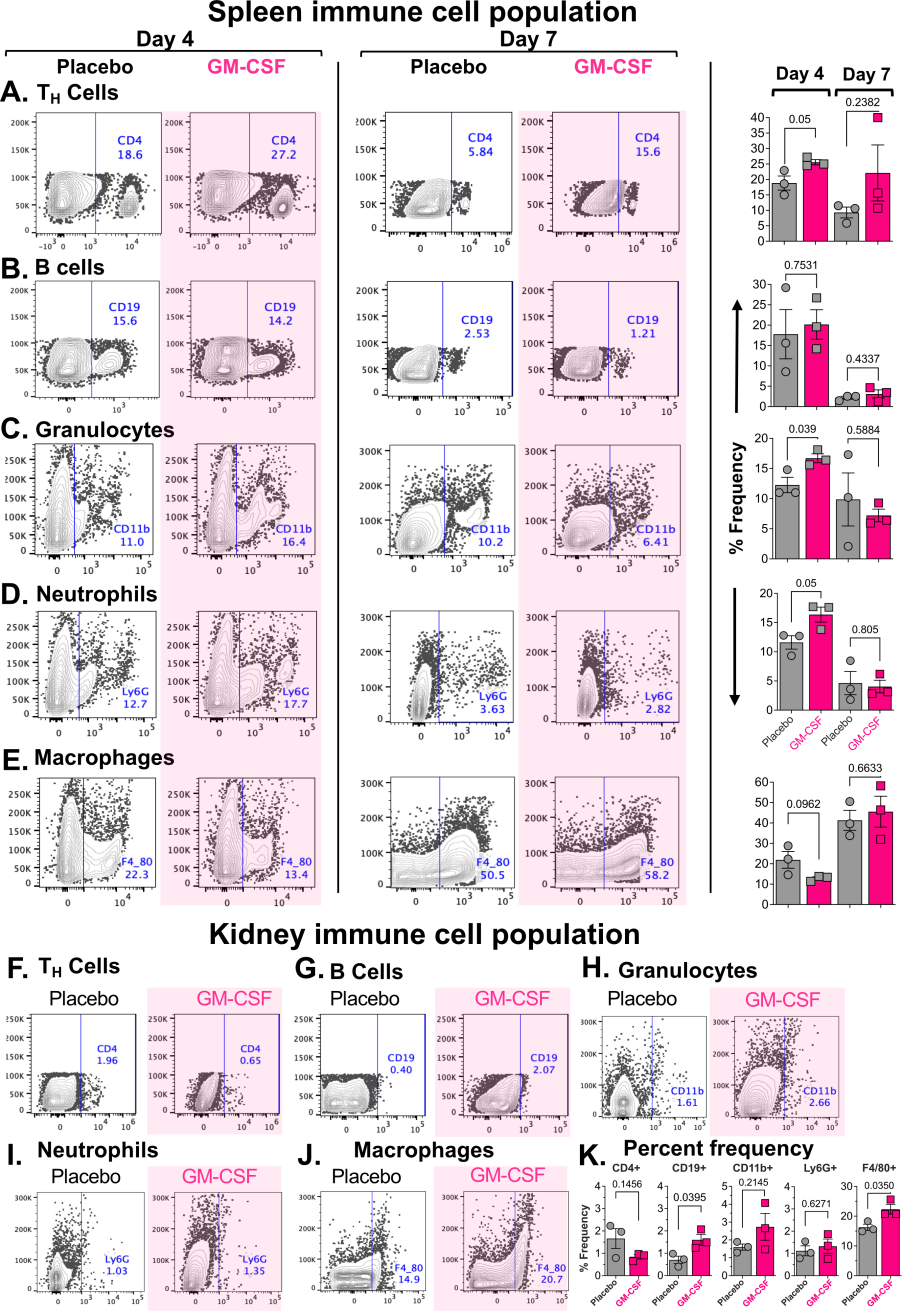
Effect of GM-CSF treatment on the spleen and kidney immune cell population in *C. auris*-infected immunosuppressed mice. Immunosuppressed ICR CD-1 mice (n=3/group) were infected with 1 × 10^7^ yeast cells/mouse of *C. auris* (CAU-09) and treated with a 2 μg/mouse dose of murine GM-CSF once daily starting 24 hours to 96 hours post-infection. Four hours after the final treatment, spleens (days 4 and 7 post-infection, **(A-E)** and kidneys (7 days post-infection, **F-K**) of the mice were processed to prepare a single-cell suspension and stained with fluorescently labeled antibodies for specific immune cell lineage markers: CD4 for T helper cells **(A, F)**, CD19 for B cells **(B, G)**, CD11b for Granulocytes **(C, H)**, Ly6G for Neutrophils **(D, I)**, and F4/80 for Macrophages **(E, J)**. Isotype-matched antibodies were used as controls for gating the negative and positive populations. The stained cells were analyzed using a BD FACSymphony, and the data were analyzed with FlowJo v10. Representative scatter graphs and corresponding bar graphs (mean ± SEM) are shown for the placebo and GM-CSF treatment groups **(K)**. Significant differences in immune cell populations were analyzed using Student’s t-tests (two-tailed), and p-values <0.05 were considered statistically significant.

Next, we investigated the effect of GM-CSF treatment on the frequency of different immune cell subsets in the spleen on days 4 and 7 after *C. auris* infection using flow cytometry. GM-CSF treatment resulted in a strong trend of elevated CD4^+^ T helper cell frequency on day 4 post-infection vs placebo treatment (mean difference: 6.733, 95% CI: -0.01190 – 13.48, two-tailed t-test p= 0.0503), but this trend was diminished by day 7 (mean difference: 12.79, 95% CI: -12.84 – 38.41, two-tailed t-test p = 0.2382, **Figures 4A**, **Supplementary Figure S4A**). B Cell frequencies were similar in both the placebo and GM-CSF-treated mice after days 4 and 7 post-infections (**Figure 4B**, **Supplementary Figure S4B**). The CD11b^+^ granulocytes have a significantly elevated frequency in GM-CSF-treated mice vs placebo-treated mice (mean difference: 4.433, 95% CI: 0.3644 – 8.502, two-tailed t-test p = 0.039) and a strong trend of elevated neutrophils (Ly6G^+^) frequency (mean difference: 4.763, 95% CI: -0.01623 – 9.543, two-tailed t-test p = 0.0505) on day 4 post-infection, but not on day 7 (**Figures 4C, D**; **Supplementary Figures S4C, D**). The macrophages (F4/80^+^) were not altered by GM-CSF treatment compared to the placebo on either day 4 or 7 post-infection (**Figures 4E**, **Supplementary Figure S4E**). Notably, T helper cells, B cells, granulocytes, and neutrophils showed a decrease in the spleen by day 7 in both treatment groups, while macrophage populations were significantly increased.

We also determined the frequencies of immune cells in the kidneys of immunosuppressed mice after 7 days of *C. auris* infection, the primary target organ in this model. GM-CSF-treated mice showed a significant increase in the B cell (mean difference: 0.8967, 95% CI: 0.07003 – 1.723, two-tailed t-test p = 0.0395) and macrophage (mean difference: 6.067, 95% CI: 0.6957 – 11.44, two-tailed t-test p = 0.035) populations vs placebo-treated mice. In contrast, the frequencies of T helper cells, granulocytes, and neutrophils were not significantly different from those in the placebo group (**Figures 4F–K**; **Supplementary Figures S5A-E**). We did not evaluate the immune cell populations in the kidneys on day 4 post-infection. The detailed statistical description of the frequencies of immune cell phenotype is provided in **Supplementary Table S1** under the **Supplementary file**.

We also assessed the levels of proinflammatory cytokines (IFN-γ, TNF-α, IL-2, IL-4, IL-6, IL-12p70, IL-17A, and IL-10) in the spleen of placebo or GM-CSF-treated *C. auris*-infected or uninfected mice. GM-CSF-treated *C. auris*-infected mice showed significantly increased levels of IL -6 (Median: 11.64 pg/ml, IQR: 9.133 – 37.74) vs uninfected mice (Median: 7.748 pg/ml, IQR: 7.666 – 7.817, adjusted p=0.0043, **Supplementary Figure S6A**). Similarly, TNF-α levels (Median: 1.604 pg/ml, IQR: 1.497 – 1.646) were also significantly increased vs uninfected mice (Median: 1.256 pg/ml, IQR: 7.666 – 7.817, adjusted p=0.0128, **Supplementary Figure S6B**). Notably, TNF-α was detected at very low levels (<2 pg/ml), while other cytokines were undetectable.

### GM-CSF enhances neutrophil and macrophage protective responses against *C. auris*

**Figure 5:**
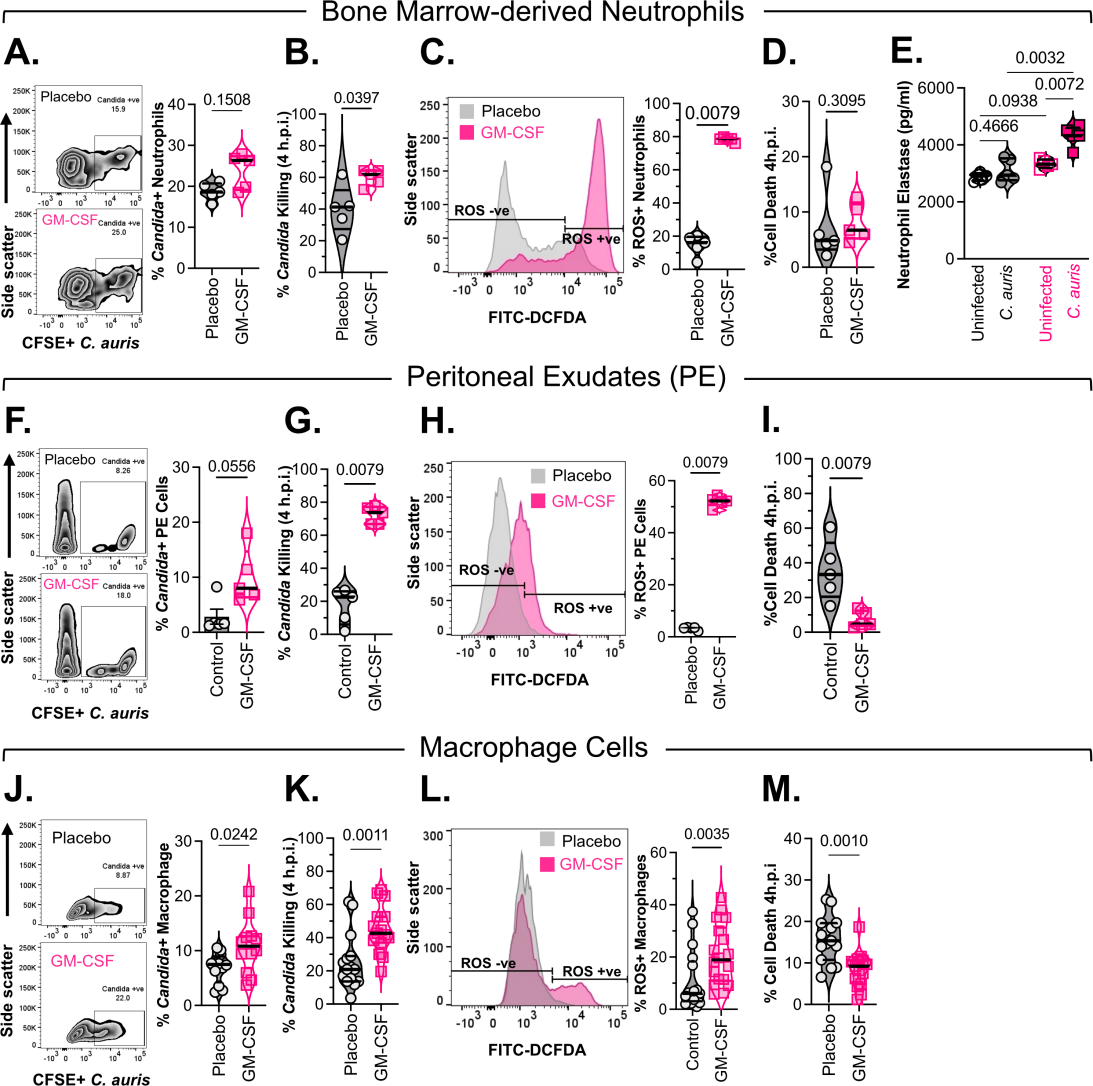
Neutrophil and Macrophage antifungal responses after GM-CSF treatment. Immunocompetent ICR CD-1 mice (n=5/group) were treated with a 2 μg/mouse dose of murine GM-CSF once daily. After 4 days of treatment, neutrophils purified from the bone marrow **(A-E)** and peritoneal exudates **(F-I)** from each mouse were cultured *ex vivo*. RAW264.7 macrophages **(J-M)** were pre-treated with murine GM-CSF (20 ng/ml) for 16 hours. 0.5–1 x 10^6^ cells were then infected with *C. auris* (MOI 1) for different time points. Representative scatter plot and corresponding bar graph (n=5 mice/group) demonstrating phagocytic uptake of CFSE-labelled *C. auris* 1.5 hours post-infection by bone marrow neutrophils **(A)**, peritoneal exudates **(F)**, and RAW264.7 macrophages **(J)**, were assessed by flow cytometry. Survival of *C. auris* in the presence or absence of neutrophils **(B)**, peritoneal exudates **(G)**, or RAW264.7 macrophages **(K)** was determined by CFU counts. The percentage killing by neutrophils was calculated thereafter presented. Reactive oxygen species (ROS) generated by neutrophils **(C)**, peritoneal cells **(H)**, or RAW264.7 macrophages **(L)** upon infection with *C. auris* 1 h post-infection were measured by DCFDA staining and flow cytometry. Pooled data from each group is plotted. Neutrophil **(D)**, peritoneal cell **(I)**, or RAW264.7 macrophages **(M)**, death 4 hours post-infection with *C. auris* was determined by measuring LDH release in the media. Secreted levels of neutrophil elastase in the supernatant, upon infection with *C. auris* 4 hours post-infection, were measured by ELISA **(E)**. All graphical data are expressed as violin plots with median ± IQR. Statistical comparisons were made using the Mann-Whitney test and calculated two-tailed exact p-value **(A-D, F-M)** or a two-way ANOVA **(E)**, followed by Tukey’s multiple-comparison test and calculated adjusted p-value. P<0.05 was considered significant.

We further investigated the immunomodulatory effects of GM-CSF treatment on *ex vivo* functional activities of immune cells and profiled immune cells from the bone marrow and peritoneal exudates of GM-CSF- or placebo-treated mice. Bone marrow neutrophils from GM-CSF-treated mice (3 out of 5) demonstrated increased phagocytic uptake of labelled *C. auris* (**Figure 5A**), along with significantly increased clearance of the fungus at four hours (U = 2.5, 2-tailed exact p=0.0397, **Figure 5B**). Phagocytic uptake of pathogens by innate immune cells is accompanied by the generation of reactive oxygen species (ROS) as an early antimicrobial response (34). Thus, we next investigated how GM-CSF treatment modulated neutrophil ROS responses during *C. auris* infection. Remarkably, *C. auris*-induced ROS was significantly higher in neutrophils from GM-CSF-treated mice compared to the placebo group (U = 0, 2-tailed exact p=0.0079, **Figure 5C**). Activated neutrophils often utilize ROS-dependent neutrophil extracellular trap formation (NETosis) for efficient clearance of pathogens (35). As a surrogate for NETosis, we measured neutrophil elastase release into the media upon *C. auris* infection. As expected, an increased *C. auris*-induced ROS in neutrophils from GM-CSF-treated mice also showed significantly increased secreted neutrophil elastase (Mean difference ± SE: 1214 ± 138.1, 2-way ANOVA with Tukey’s adjusted p=0.0032). NETosis is usually associated with increased neutrophil cell death during infection (36). However, we did not observe a significant increase in neutrophil cell death in *C. auris*-infected neutrophils derived from the bone marrow of GM-CSF-treated mice compared to placebo-treated mice (**Figures 5D, E**). A similar enhanced antifungal phenotype was also observed for cells from the peritoneal cavity, with these cells from GM-CSF-treated mice showing a strong trend in the enhancement of phagocytic uptake of fluorescently labelled *C. auris* (U = 3, 2-tailed exact p= 0.0556, **Figure 5F**), clearance of fungus (U = 0, 2-tailed exact p= 0.0079, **Figure 5G**), and generation of *C. auris*-induced ROS (U = 0, 2-tailed exact p= 0.0079, **Figure 5H**). However, unlike bone marrow neutrophils, these cells were significantly more resistant to *C. auris*-induced cell death than cells from the placebo group (U = 0, 2-tailed exact p=0.0079, **Figure 5I**).

In the absence of GM-CSF, there was negligible macrophage infiltration in the peritoneal cavity (**Supplementary Figure S3B**). Therefore, to investigate the effect of GM-CSF on macrophage antifungal responses, we used RAW264.7 macrophages. GM-CSF-treated RAW macrophages exhibited a phenotype similar to that of *ex vivo* neutrophils. An overnight treatment of GM-CSF resulted in a significantly enhanced uptake (U = 33, 2-tailed exact p=0.0242, **Figure 5J**) and clearance of *C. auris* by the GM-CSF-treated macrophages (U = 37, 2-tailed exact p=0.0011, **Figure 5K**). GM-CSF treatment also resulted in a significantly enhanced *C. auris*-induced ROS (U = 66.5, 2-tailed exact p=0.0035, **Figure 5L**). Additionally, as previously observed with cells from the peritoneal cavity, GM-CSF-treated RAW macrophages were significantly more resistant to *C. auris*-dependent cell death (U = 36, 2-tailed exact p=0.001, **Figure 5M**). The detailed statistical descriptions of these *in vitro* anti-*C. auris* immune cell functional activities are available in **Supplementary Tables S2-S3** under the **Supplementary file**.

Finally, no adverse events (e.g., weight loss, respiratory distress, behavioral changes, moribundity) were observed in uninfected immunocompetent (n=5) or immunosuppressed (n=3) mice during or after four days of GM-CSF administration (2 μg/mouse), indicating that the treatment was well tolerated under these conditions.

## Discussion

GM-CSF is a pleiotropic cytokine known to promote myelopoiesis and enhance the antimicrobial functions of myeloid cells, including monocytes, neutrophils, macrophages, and dendritic cells (37–39). Given its immunomodulatory properties, GM-CSF holds promise as a therapeutic agent, especially in immunocompromised individuals who are highly susceptible to opportunistic fungal infections (40–42). This immunomodulatory effect is particularly relevant for *C. auris*, which is not only highly drug-resistant but also difficult to eliminate (43). Moreover, *C. auris* infections are associated with alarmingly high mortality rates in immunosuppressed patients (1, 44), predominantly due to limited antifungal treatment options and to the underlying defects in host immune defense (1, 45–47). Together, these pose a significant challenge in the management of *C. auris* in clinical settings. In our study, we investigated the therapeutic efficacy of GM-CSF in a clinically relevant murine model of hematogenously disseminated *C. auris* infection. GM-CSF treatment enhanced immune cell populations and their antifungal responses, resulting in improved survival and reduced disease severity, thereby supporting its potential as an adjunctive immunotherapeutic strategy.

Our findings further underscore the clinical relevance of GM-CSF, particularly in disseminated *C. auris* infection under immunosuppressed conditions. In our murine model, GM-CSF monotherapy significantly improved survival outcomes and mitigated disease severity. Notably, when combined with a subtherapeutic dose of micafungin, GM-CSF conferred even greater protection, increasing survival to 56% compared with 0% in the placebo group. Thus, GM-CSF and micafungin did not show any antagonistic effect, and, in fact, slightly increased survival compared to monotherapy. This proof-of-concept study highlights the potential of GM-CSF as an effective adjunct therapy that can be administered alongside therapeutic antifungal treatment in clinical settings.

Additionally, GM-CSF treatment alleviated weight loss and markedly reduced fungal burden and tissue damage in the kidney, heart, and brain. GM-CSF treatment failed to prevent mortality but did prevent weight loss in the immunosuppressed *C. albicans* infection model, potentially because *C. albicans* is more virulent than *C. auris*, which can elicit exaggerated immune responses. Alternatively, this difference could reflect fundamental differences in host-pathogen interactions and immune modulation strategies between the two *Candida* species. These findings highlight the need for species-specific strategies when considering GM-CSF as an adjunctive therapy.

In uninfected immunosuppressed mice, GM-CSF administration led to a marked increase in splenic CD3^+^ T cells, CD4^+^ T helper cells, and CD11b^+^ granulocytes. In contrast, B cell populations showed a non-significant downward trend. Interestingly, neutrophil frequencies in the spleen and bone marrow were elevated, but macrophage populations remained unchanged. These findings indicate that GM-CSF selectively promotes expansion of key innate and adaptive immune subsets, particularly those involved in antifungal defense. During early *C. auris* infection (day 4 post-infection), GM-CSF treatment significantly increased granulocyte frequencies and showed trends toward elevated T helper cell and neutrophil frequencies in the spleen. However, by day 7, these populations declined in both treated and control groups, likely reflecting the onset of regulatory immune responses, immune exhaustion, or pathogen-induced immunosuppression. Notably, macrophage frequencies increased at this later time point, suggesting a delayed compensatory response. In the kidneys, GM-CSF treatment significantly elevated B cell and macrophage populations, highlighting tissue-specific immune modulation. These dynamic changes underscore the temporal and compartmentalized nature of GM-CSF’s immunomodulatory effects during disseminated fungal infection. The increase in immune cell populations, particularly neutrophils, upon GM-CSF administration is consistent with previous reports (48–53). Furthermore, an increase in the lymphocyte population in humans after GM-CSF therapy has been previously reported (48). A GM-CSF–IRF5 signaling axis through eosinophils has been reported to promote T cell activation and tumor infiltration, particularly CD4 Th1 cells, and may be responsible for the increased frequency of these cells in our study (54).

In addition to immune cell profiling, we assessed proinflammatory cytokine profiles in GM-CSF-treated mice. We found significantly elevated IL-6 levels in *C. auris*-infected mice vs uninfected mice treated with GM-CSF, likely due to inflammation driving B and T cell proliferation and activation (53, 55–57). We also detected very low levels of TNF-α expression in the spleens of infected mice, with a significant increase in *C. auris*-infected mice regardless of GM-CSF treatment. The lack of detection of other cytokines may reflect the unique immune-evasion properties of *C. auris*, or the sampling timing may have been closer to immunosuppressed state of the mice.

We also observed an increase in neutrophil and macrophage populations in the peritoneal cavity after GM-CSF treatment in uninfected immunocompetent mice, further supporting its role in trafficking immune cells across different compartments (41, 42). Bone marrow neutrophils from these GM-CSF-treated mice exhibited increased phagocytic uptake of *C. auris*, enhanced fungal clearance, and elevated ROS production. These responses were accompanied by increased neutrophil elastase release, indicative of NETosis, and a modest rise in infection-induced immune cell death. Peritoneal immune cells similarly demonstrated enhanced phagocytosis, fungal killing, and ROS generation, but were notably more resistant to *C. auris*-induced cytotoxicity. Similarly, RAW264.7 macrophages treated with GM-CSF also showed improved uptake, ROS production, and fungal clearance, along with increased resistance to cell death.

In clinical settings, GM-CSF has been employed to enhance myeloid recovery following chemotherapy and bone marrow transplantation, particularly in immunosuppressed patients (58). Clinical studies have demonstrated that recombinant GM-CSF can enhance host immune responses, improve fungal clearance, and reduce disease severity, particularly in patients with compromised immunity (18, 50, 59). For instance, GM-CSF has been used successfully as an adjunct to fluconazole in treating refractory mucosal candidiasis in AIDS patients (60). These findings align with our study, which highlights GM-CSF’s ability to traffic and activate key immune cell populations in key organs, enhance cytokine responses, and improve functional antifungal activity.

**Figure 6:**
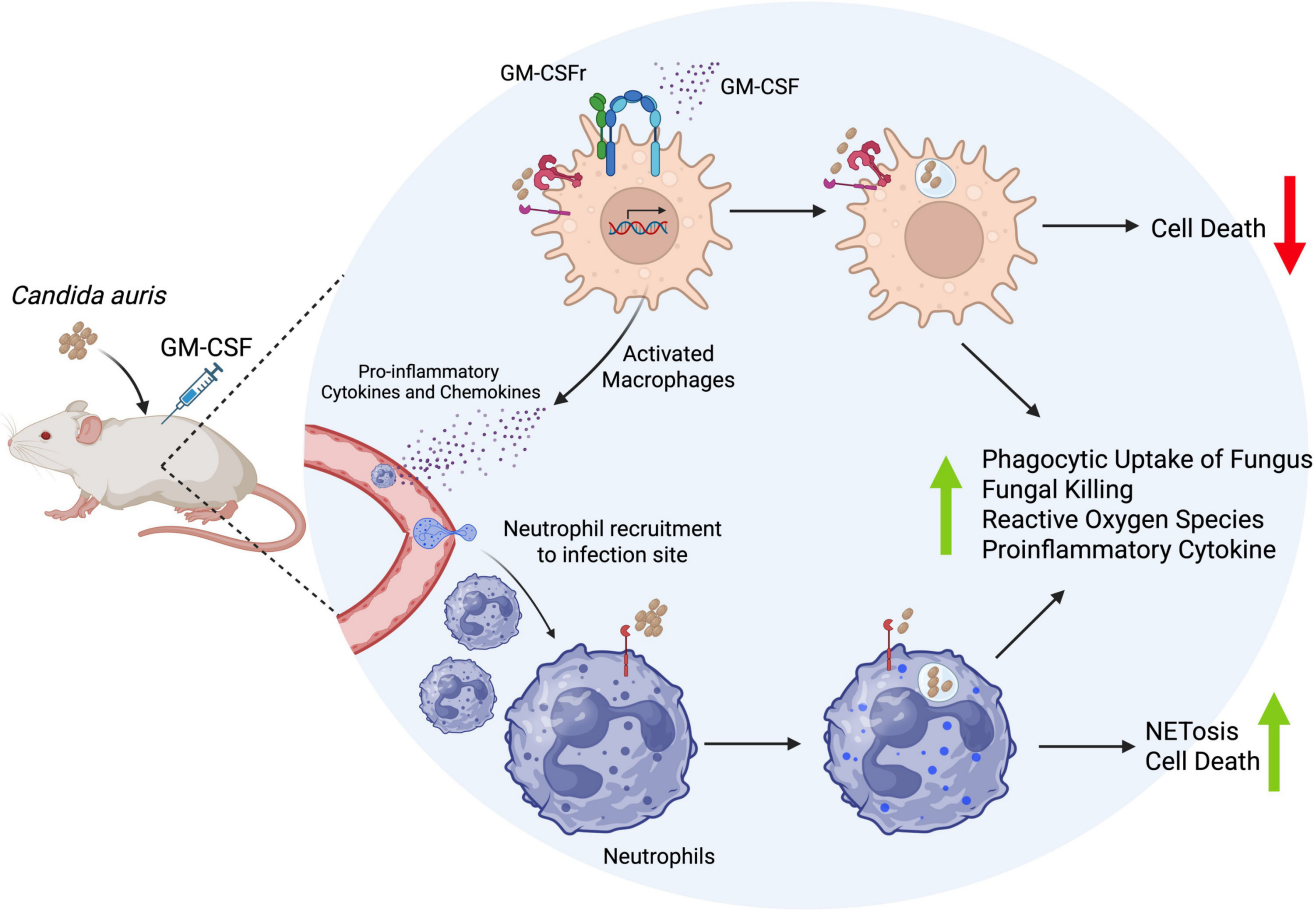
Immune modulation and antifungal activity of GM-CSF therapy against MDR *C. auris*. Exogenous GM-CSF treatment in *C. auris*-infected mice modulates immune cells by augmenting their proliferation, recruitment, and antifungal activity, particularly neutrophils and macrophages, likely via the release of macrophage-secreted proinflammatory cytokines and chemokines. Specifically, GM-CSF therapy enhances fungal uptake, NETosis, fungal killing, and the generation of Reactive Oxygen Species (ROS) by phagocytes and may also promote the secretion of a proinflammatory cytokine (IL-6), thereby reducing *the C. auris* burden in infected tissues. (Created using https://biorender.com).

Collectively, our findings underscore the therapeutic potential of GM-CSF as an adjunctive immunotherapy for multidrug-resistant *C. auris*, particularly in immunocompromised individuals, where available antifungal treatments often prove inadequate. Under conditions of immunosuppression, GM-CSF demonstrated a protective effect against lethal invasive infection, likely mediated through the expansion and activation of lymphocytes, neutrophils, and macrophages, enhancing their antifungal efficacy, as depicted in **Figure 6**.

One limitation of our study is that both *ex vivo* and *in vivo* efficacy testing focused on only *C. auris* CAU-09 strain. However, this strain is MDR and highly virulent, and the murine models represent a severe, rapidly progressing systemic infection. Our results confirm the therapeutic benefits of GM-CSF in improving overall health outcomes and position it as a promising candidate for adjunctive therapy in the management of lethal MDR *C. auris* fungal infections, particularly in immunocompromised patients. These findings are in line with prior reports showing protective benefits of GM-CSF therapy against a broad spectrum of fungal pathogens, including *Cryptococcus* spp., *Aspergillus* spp., and dimorphic fungi such as *Histoplasma capsulatum* and *Blastomyces dermatitidis* (39).

Despite its potential benefits, the use of GM-CSF as an antifungal therapy is not without challenges. One significant concern is the risk of exacerbating inflammatory responses, which could lead to complications such as cytokine release syndrome. Although no adverse events were observed in uninfected immunosuppressed or immunocompetent mice during four days of GM-CSF treatment for immune cell characterization, further comprehensive dose-ranging safety and efficacy studies are needed. These investigations should include testing additional *C. auris* isolates from different clades and immunocompetent infection models. Additionally, further research is required to establish standardized dosing protocols and identify which patient populations would benefit most from this therapy. Ultimately, clinical trials are necessary to fully evaluate its efficacy and limitations in fungal infections, particularly against MDR *C. auris*.

## Data Availability

The original contributions presented in the study are included in the article/**Supplementary Material**. Further inquiries can be directed to the corresponding author.
