# Supplementary material for: Adjunctive GM-CSF therapy enhances host defense against systemic Candida auris infection in immunosuppressed mice
Source: Front Immunol. 2026 Jan 19;16:1731315. doi: 10.3389/fimmu.2025.1731315 (PMC12862068; doi:10.3389/fimmu.2025.1731315)
Supplement: Supplementary file 1 [file DataSheet1.pdf]

Supplementary Data

Adjunctive GM-CSF Therapy Enhances Host Defense Against Systemic *Candida auris* Infection in Immunosuppressed Mice

Eliciane Cevolani Mattos[1#], Kaustav Das Gupta[1#], Derek Quintanilla[1], Haley Hautau[1], Ashraf S. Ibrahim[1,2], Shakti Singh[1,2*]

[1]Division of Infectious Disease, The Lundquist Institute for Biomedical Innovation at Harbor–University of California, Los Angeles Medical Center, Torrance, CA, USA.

[2]David Geffen School of Medicine, University of California, Los Angeles, Los Angeles, CA, USA.

# Equal contribution

*Correspondence to Shakti Singh (shakti.singh@lundquist.org)

Supplementary Data

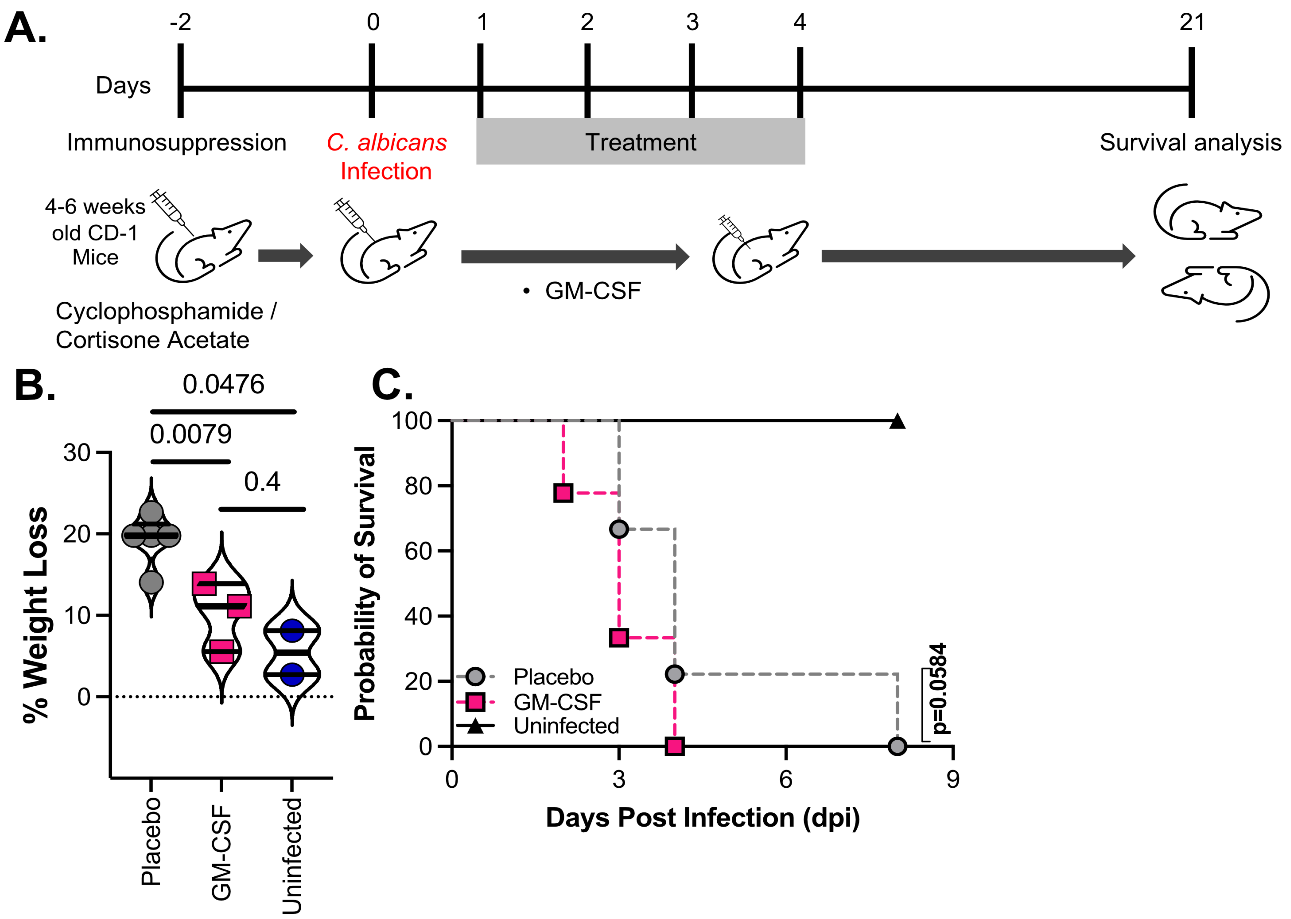

Weight loss comparison

Groups	N at day 0	N at day 3	% Weight Loss Median (Q1, Q3)	Hodges-Lehmann difference	Mann Whitney U	Two-tailed p-values (Exact)
Placebo	9	5	19.77 (16.91, 21.2)			
GM-CSF	9	3	11.11 (5.556, 13.89)	-8.66	0	0.0179
Un-infected	2	2	5.405 (2.703, 8.108)			

Survival analysis (Mantel-Cox Comparison)

Groups	Mice / Group, N		Survival at Day 8 (Endpoint)	Median Survival Time (MST in Days)		Log rank Hazard Ratio (Treatment/ Placebo)		Mantel-Cox Comparison Test, df=1	
	Day 0	Day 8	% Survival	MST	95% CI	HR	95% CI	Chi square	p-value
Placebo	9	0	0%	4	3.00 to >8				
GM-CSF	9	0	0%	3	2.00 to >8	1.814	0.6911 to 4.763	3.582	0.0584
Un-infected	2	2	100%	>8					

Figure S1. Efficacy of GM-CSF therapy against lethal *C. albicans* systemic infection in immunosuppressed mice. A). 4-6 weeks old immunosuppressed ICR CD-1 mice (n =9/group) were infected with *C. albicans* SC5314 (5 x 10^4 cells/mouse) and treated with murine 2.0 µg GM-CSF/mouse through daily intraperitoneal injections. The GM-CSF treatments started on 24 h post-infection and continued up to days 4 post-infection. **B**). % weight loss in surviving mice was compared between different treatment groups at day 3 post-infection using Mann-Whitney U test and expressed as median ± IQR (Exact, 2-tailed p-values). **C**). Mice survival was compared after 8 days post-infection by the Mantel-Cox test. P values >0.05 considered significant.

Supplementary Data

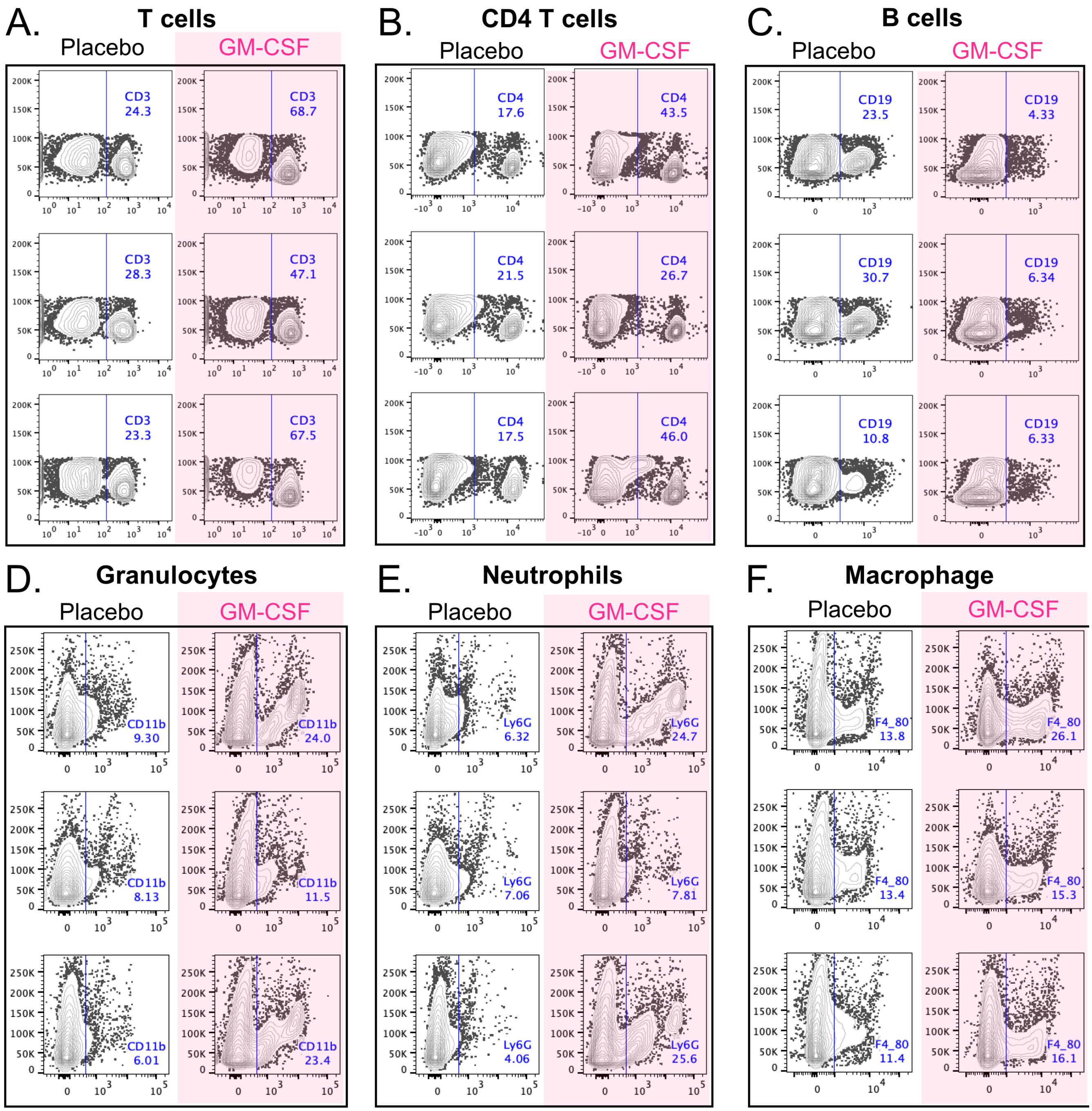

Figure S2. Effect of GM-CSF treatment on the spleen immune cell population in uninfected mice. Individual scatter plots of Spleen samples stained for markers: **A).** CD3 for total T cells, **B).** CD4 for T helper cells, **C).** CD19 for B cells, **D).** CD11b for Granulocytes, **E).** Ly6G for Neutrophils, and **F).** F4/80 for Macrophages.

Supplementary Data

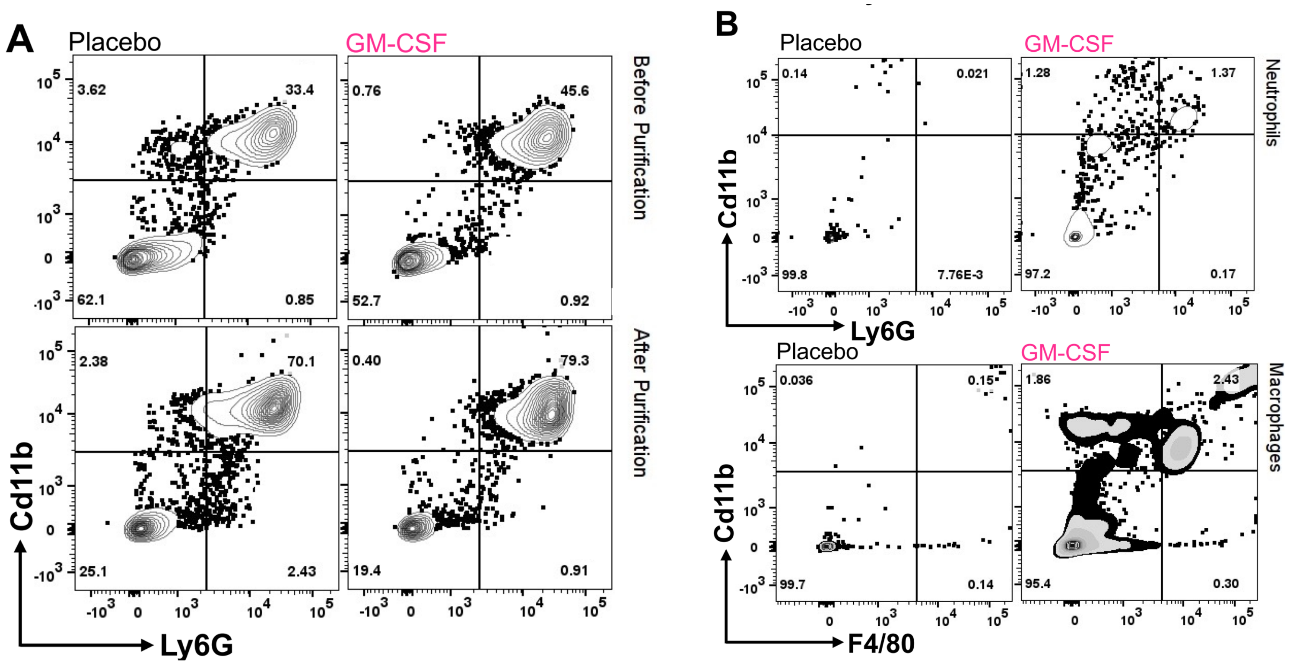

Figure S3. Bone marrow neutrophil and intraperitoneal neutrophil and macrophage population in uninfected GM-CSF-treated mice. Immunocompetent ICR CD-1 mice (n=5/group) were treated with 2 µg/mouse dose of murine GM-CSF once daily. **A).** After 4 days of treatment, bone marrow neutrophils were purified from each mouse. Purity was assessed by flow cytometry by staining the single cell suspension with antibodies against CD11b and Ly6G. Representative scattered dot plot demonstrates the degree of purification. **B).** Cells from the peritoneal cavity were also stained using antibodies against CD11b, F4/80 and Ly6G. Representative scattered dot plot demonstrates the percentage of neutrophils (CD11b$^+$ Ly6G$^+$) and macrophages (CD11b$^+$ F4/80$^+$) infiltration in the peritoneal cavity of treated mice.

Supplementary Data

A. T_H Cells

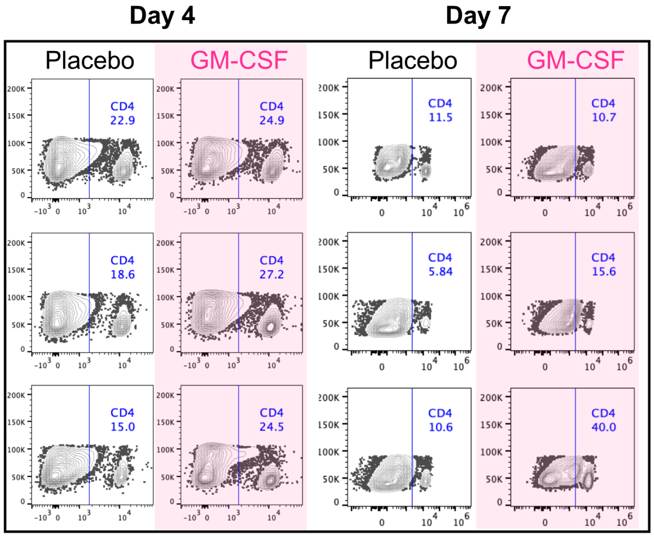

B. B Cells

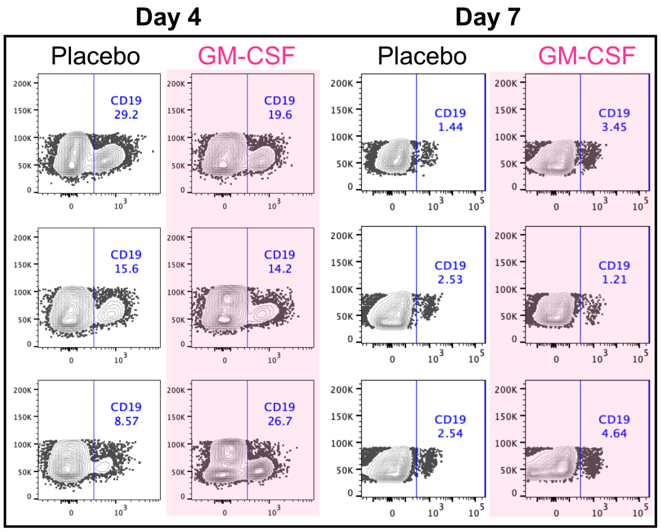

C. Granulocytes

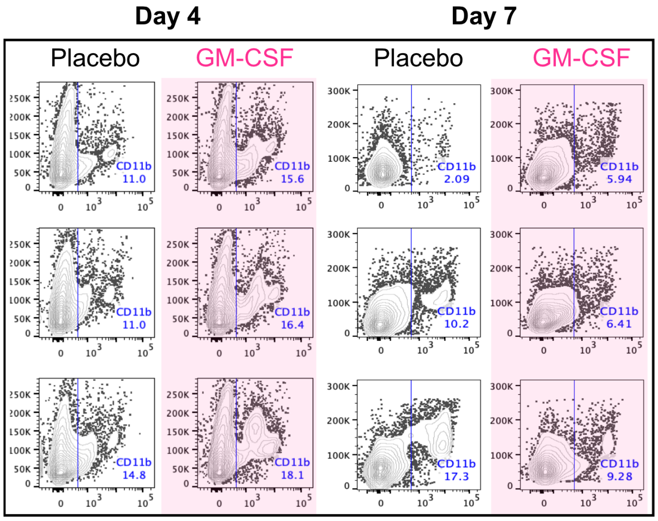

D. Neutrophils

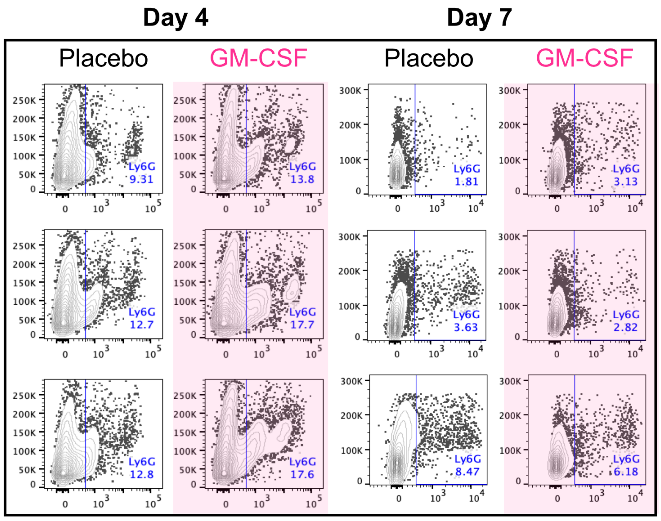

E. Macrophages

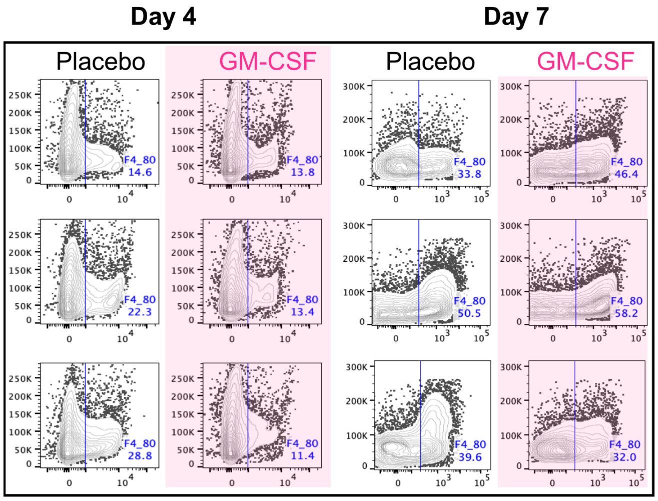

Figure S4. Effect of GM-CSF treatment on the spleen immune cell population in *C. auris*-infected mice. The *C. auris*-infected immunosuppressed mice were treated with a placebo or GM-CSF starting 24 h to 96 h post-infection, and spleen immune cell populations were analyzed on days 4 and 7 post-infection. Individual scatter plots of spleen samples stained for markers: **A).** CD4 for T helper cells, **B).** CD19 for B cells, **C).** CD11b for Granulocytes, **D).** Ly6G for Neutrophils, and **E).** F4/80 for Macrophages.

Supplementary Data

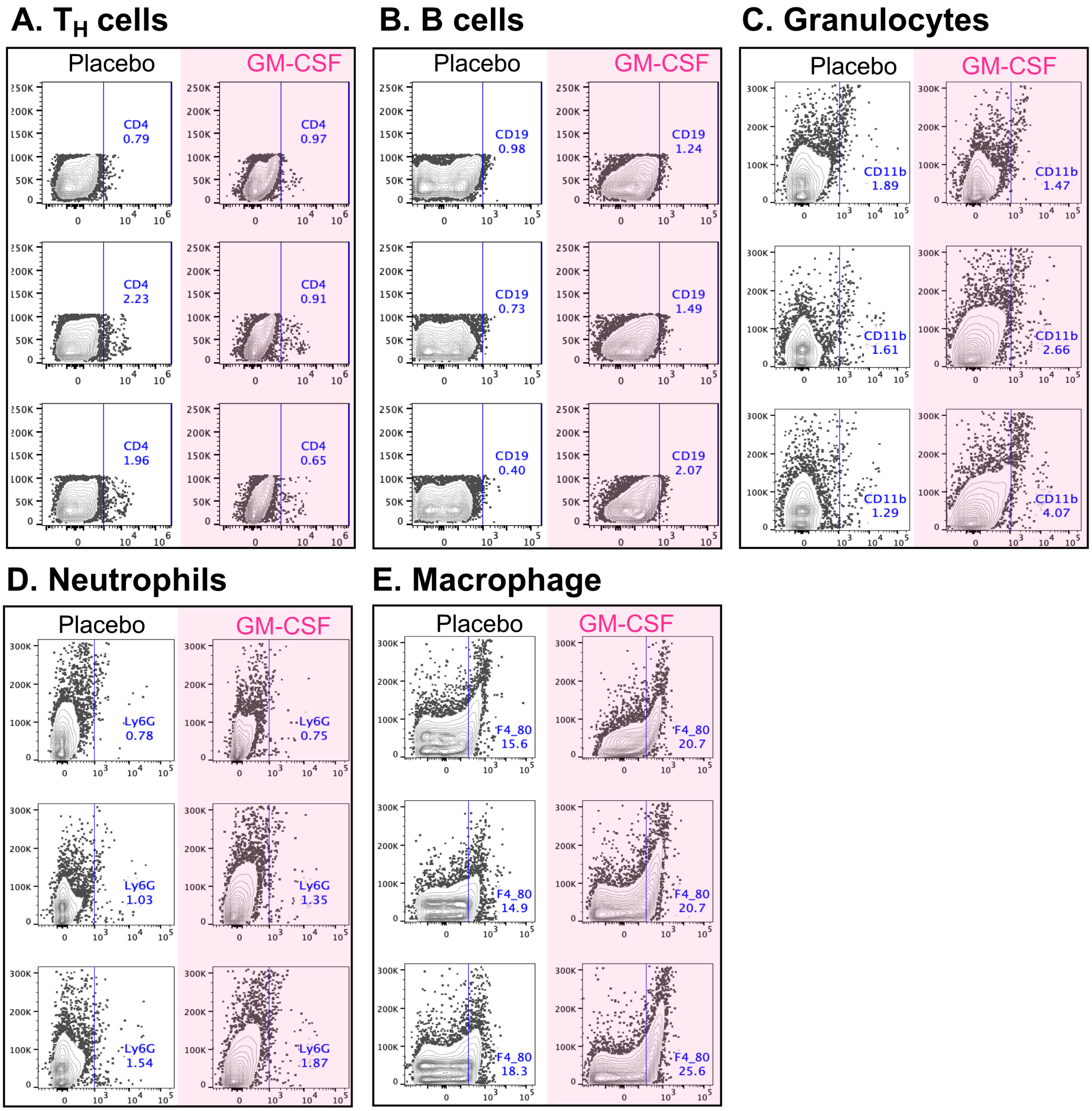

Figure S5. Effect of GM-CSF treatment on the kidney immune cell population in *C. auris*-infected immunosuppressed mice. The *C. auris*-infected immunosuppressed mice were treated with a placebo or GM-CSF starting 24 h to 96 h post-infection, and spleen immune cell populations were analyzed on day 7 post-infection. Individual scatter plots of Kidney samples stained for markers: **A).** CD4 for T helper cells, **B).** CD19 for B cells, **C).** CD11b for Granulocytes, **D).** Ly6G for Neutrophils, and **E).** F4/80 for Macrophages.

Supplementary Data

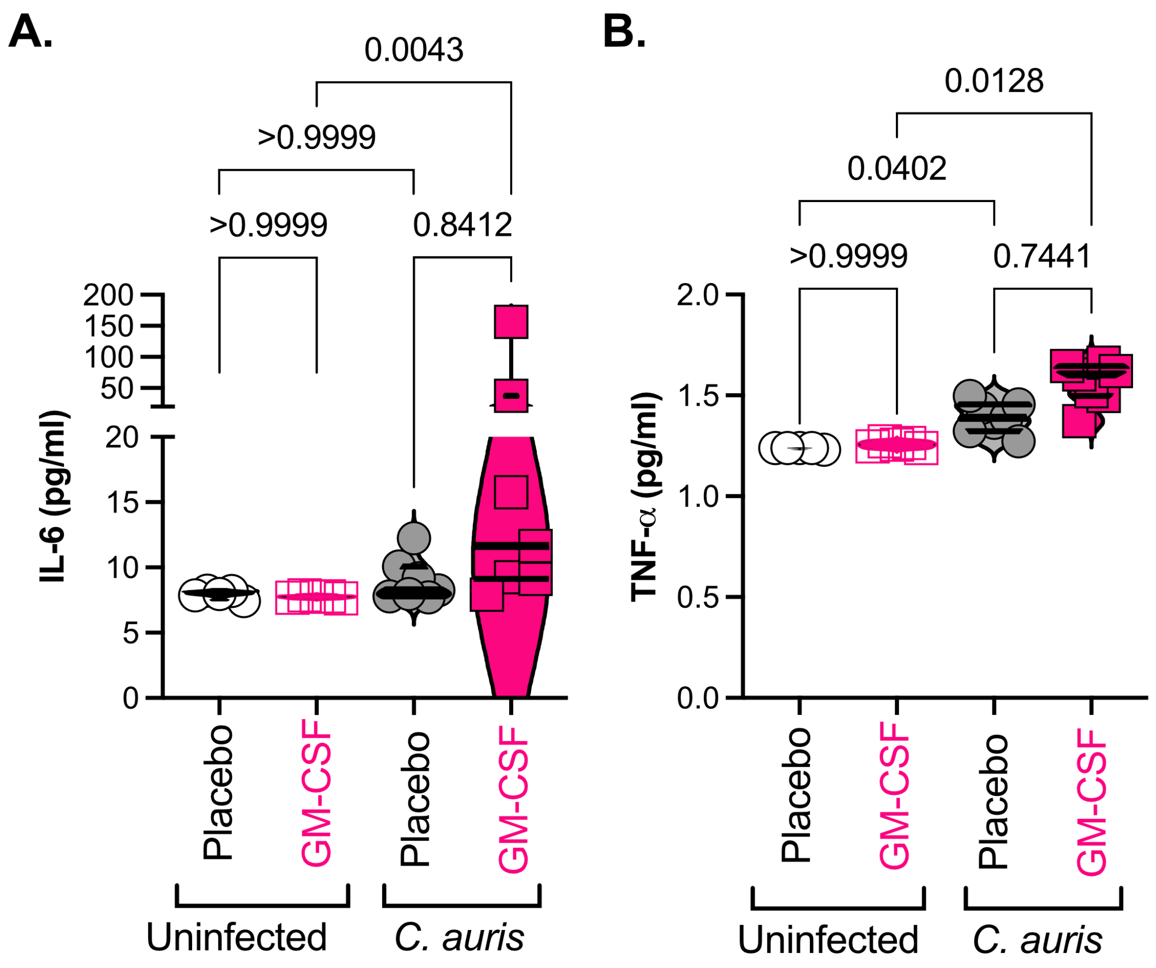

Descriptive analysis of cytokines

Groups	N	IL-6 pg/ml		TNF-alpha pg/ml	
		Mean ± SE	Median (Q1, Q3)	Mean ± SE	Median (Q1, Q3)
**Uninfected: Placebo**	5	7.93 ± 0.1446	7.968 (7.638, 8.203)	1.237 ± 0.0016	1.239 (1.235, 1.239)
**Uninfected: GM-CSF**	5	7.742 ± 0.03728	7.748 (7.666, 7.817)	1.256 ± 0.005941	1.256 (1.244, 1.269)
***C. auris*: Placebo**	7	9.032 ± 0.6268	8.244 (7.775, 10.09)	1.391 ± 0.0297	1.389 (1.322, 1.455)
***C. auris*: GM-CSF**	7	35.39 ± 20.53	11.64 (9.133, 37.74)	1.561 ± 0.03919	1.604 (1.497, 1.646)

Figure S6. Spleen cytokine analysis of uninfected and *C. auris*-infected mice treated with GM-CSF. Immunosuppressed ICR CD-1 mice were either uninfected (n=5/group) or infected (n=7/group) with 1 x 10^7 *C. auris* (CAU-09) cells/mouse. The mice were treated with 2 µg/mouse dose of murine GM-CSF once daily for 4 days. After 4 days, the mice's spleens were processed to generate lysates. Splenic concentration of IL-6 **(A)** and TNF-α **(B)** was then measured using the Mouse Magnetic Luminex Assay Kit and presented as median ± IQR. Significant differences in treatment groups were analyzed by Kruskal-Wallis test followed by Dunn's correction for multiple comparison. Adjusted P-values <0.05 were considered statistically significant.

Supplementary Data

Table S1. Immune cell frequency in placebo vs GM-CSF-treated mice

Panel	Immune Cell	Mean ± SE (N= 3 mice /group)		Mean difference ± SE	95% CI	p-values (Two-tailed t-test)
		Placebo	GM-CSF			
**Immune cell frequency in the <u>spleen of uninfected</u> GM-CSF vs Placebo-treated mice (Figure 3, S2)**						
**A**	T cells	25.30 ± 1.528	61.10 ± 7.009	35.80 ± 7.173	15.88 to 55.72	0.0075
**B**	T_H cells	18.87 ± 1.317	38.73 ± 6.060	19.87 ± 6.201	2.649 to 37.08	0.0328
**C**	B cells	21.67 ± 5.817	5.667 ± 0.6683	-16.00 ± 5.856	-32.26 to 0.2577	0.0523
**D**	Granulocytes	7.813 ± 0.9628	19.63 ± 4.07	11.82 ± 4.183	0.207 to 23.43	0.0475
**E**	Neutrophils	5.813 ± 0.9023	19.67 ± 5.84	13.85 ± 5.910	-2.555 to 30.26	0.079
**F**	Macrophages	19.17 ± 3.474	22.03 ± 3.043	2.867 ± 4.618	-9.956 to 15.69	0.5684
**Immune cell frequency in the <u>spleen</u> of GM-CSF vs Placebo-treated mice at <u>day 4 post *C. auris* infection</u> (Figure 4A-E, S4A-E)**						
**A**	T_H cells	18.83 ± 2.284	25.57 ± 0.8293	6.733 ± 2.429	-0.01190 to 13.48	0.0503
**B**	B cells	17.79 ± 6.055	20.17 ± 3.62	2.377 ± 7.055	-17.21 to 21.96	0.7531
**C**	Granulocytes	12.27 ± 1.267	16.7 ± 0.7371	4.433 ± 1.466	0.3644 to 8.502	0.039
**D**	Neutrophils	11.6 ± 1.147	16.37 ± 1.284	4.763 ± 1.721	-0.01623 to 9.543	0.0505
**E**	Macrophages	21.9 ± 4.104	12.87 ± 0.7424	-9.033 ± 4.171	-20.61 to 2.546	0.0962
**Immune cell frequency in the <u>spleen</u> of GM-CSF vs Placebo-treated mice at <u>day 7 post *C. auris* infection</u> (Figure 4A-E, S4A-E)**						
**A**	T_H cells	9.313 ± 1.756	22.1 ± 9.061	12.79 ± 9.230	-12.84 to 38.41	0.2382
**B**	B cells	2.17 ± 0.365	3.1 ± 1.006	0.9300 ± 1.070	-2.040 to 3.900	0.4337
**C**	Granulocytes	9.863 ± 4.394	7.21 ± 1.044	-2.653 ± 4.516	-15.19 to 9.886	0.5884
**D**	Neutrophils	4.637 ± 1.987	4.043 ± 1.072	-0.5933 ± 2.258	-6.863 to 5.676	0.8057
**E**	Macrophages	41.3 ± 4.895	45.53 ± 7.576	4.233 ± 9.020	-20.81 to 29.28	0.6633
**Immune cell frequency in the <u>kidney</u> of GM-CSF vs Placebo-treated mice at <u>day 7 post *C. auris* infection</u> (Figure 4F-K, S5A-E)**						
**A**	T_H cells	1.66 ± 0.4419	0.8433 ± 0.09821	-0.8167 ± 0.4527	-2.074 to 0.4403	0.1456
**B**	B cells	0.7033 ± 0.168	1.6 ± 0.2458	0.8967 ± 0.2977	0.07003 to 1.723	0.0395
**C**	Granulocytes	1.597 ± 0.1733	2.733 ± 0.7515	1.137 ± 0.7712	-1.004 to 3.278	0.2145
**D**	Neutrophils	1.117 ± 0.2236	1.323 ± 0.3236	0.2067 ± 0.3933	-0.8854 to 1.299	0.6271
**E**	Macrophages	16.27 ± 1.037	22.33 ± 1.633	6.067 ± 1.934	0.6957 to 11.44	0.035

Supplementary Data

Table S2. Functional immune activity against *C. auris* in response to GM-CSF treatment

Figure 5 Panel	Immune Function	Groups	N	Mean ± SE	Median (Q1, Q3)	Hodges-Lehmann difference	Mann Whitney U	2-tailed p-values (Exact)
A	% *Candida* +ve neutrophils	Placebo	5	18.72 ± 0.9646	18.6 (16.80, 20.70)	6.100	5	0.1508
		GM-CSF	5	23.86 ± 1.951	26.3 (19.15, 27.35)			
B	% *Candida* killing by neutrophils	Placebo	5	40.00 ± 6.690	41.27 (27.38, 51.98)	22.23	2.5	0.0397
		GM-CSF	5	60.00 ± 2.309	61.90 (54.76, 64.29)			
C	% ROS +ve Neutrophils	Placebo	5	14.57 ± 2.857	16.10 (8.730, 19.65)	62.60	0	0.0079
		GM-CSF	5	78.34 ± 0.6282	78.70 (77.25, 79.25)			
D	% Neutrophil cell death	Placebo	5	7.059 ± 2.847	4.813 (3.209, 12.03)	1.904	7	0.3095
		GM-CSF	5	8.060 ± 1.518	6.716 (5.224, 11.57)			
F	% *Candida* +ve Peritoneal Exudates cells	Placebo	5	2.846 ± 1.356	1.610 (1.340, 4.970)	6.340	3	0.0556
		GM-CSF	5	10.03 ± 2.199	8.020 (6.370, 14.70)			
G	% *Candida* killing by Peritoneal Exudates cells	Placebo	5	17.33 ± 4.857	22.58 (5.991, 26.04)	52.53	0	0.0079
		GM-CSF	5	72.35 ± 2.354	73.73 (66.82, 77.19)			
H	% ROS+ Peritoneal Exudates cells	Placebo	5	3.210 ± 0.2796	3.360 (2.725, 3.620)	48.87	0	0.0079
		GM-CSF	5	51.96 ± 0.9479	52.20 (50.15, 53.65)			
I	% Peritoneal Exudates cells death	Placebo	5	35.45 ± 7.720	33.33 (20.45, 51.52)	-28.66	0	0.0079
		GM-CSF	5	7.339 ± 2.132	4.587 (3.670, 12.39)			
J	% *Candida* +ve macrophages	Placebo	12	6.850 ± 0.7799	7.495 (4.233, 8.930)	3.320	33	0.0242
		GM-CSF	12	10.81 ± 1.409	10.80 (6.825, 12.63)			
K	% *Candida* killing by macrophages	Placebo	15	25.56 ± 4.323	20.91 (13.64, 28.95)	21.82	37	0.0011
		GM-CSF	15	45.04 ± 3.701	42.73 (37.05, 52.63)			
L	% ROS +ve macrophages	Placebo	17	10.81 ± 2.745	6.080 (3.055, 18.25)	9.965	66.50	0.0035
		GM-CSF	18	20.76 ± 2.723	18.95 (10.91, 31.28)			
M	% macrophage cell death	Placebo	15	15.70 ± 1.429	15.37 (10.75, 19.62)	-7.484	36	0.0010
		GM-CSF	15	8.406 ± 1.112	9.220 (4.965, 10.64)			

Table S3. Elastase activity in response to GM-CSF treatment (Figure 5E)

2-Way ANOVA (Tukey's multiple comparisons test)	N per group	Mean 1	Mean 2	Mean difference	SE of difference	95% CI of difference	Adjusted P Value
Placebo: Uninfected vs. Placebo: *C. auris*	5	2880	3102	-221.8	138.1	-784.0 to 340.4	0.4666
Placebo: Uninfected vs. GM-CSF: Uninfected	5	2880	3338	-457.8	138.1	-1020 to 104.4	0.0938
Placebo: Uninfected vs. GM-CSF: *C. auris*	5	2880	4316	-1436	138.1	-1998 to -873.4	0.0017
Placebo: *C. auris* vs. GM-CSF: Uninfected	5	3102	3338	-236	138.1	-798.2 to 326.2	0.4244
Placebo: *C. auris* vs. GM-CSF: *C. auris*	5	3102	4316	-1214	138.1	-1776 to -651.6	0.0032
GM-CSF: Uninfected vs. GM-CSF: *C. auris*	5	3338	4316	-977.8	138.1	-1540 to -415.6	0.0072

Supplementary Data

Table S4. Fluorescent antibody used in this study

Antibody / Fluorescent Probes	Clone	Vendor	Catalog Number
CD3-Alexa Fluor700	17A2	Fisher Scientific	50-168-42
CD4-PE	CT-CD4	Fisher Scientific	PIMA517450
CD19-FITC	eBio1D3	Fisher Scientific	50-947-1
F4/80-APC	BM8	Fisher Scientific	50-112-9524
Ly6G-PE	1A8	Fisher Scientific	50-112-2246
CD11b-FITC	M1/70	Fisher Scientific	50-112-9521
Propidium Iodide (Live/Dead)		Fisher Scientific	P3566
IgG2a Kappa Isotype-PE	eBR2a	Fisher Scientific	50-186-44
IgG2a Kappa Isotype-FITC	eBR2a	Fisher Scientific	50-186-05
IgG2a Kappa Isotype-APC	eBR2a	Fisher Scientific	50-112-9530
IgG2b Kappa Isotype- Alexa Fluor700	KLH/G2b-1-2	Southern Biotech	0118-27
